# Classification and regulatory interactions of key transcription factors in COVID-19

**DOI:** 10.3389/fcimb.2025.1645333

**Published:** 2025-09-30

**Authors:** Ndimo Modipane, Saidon Mbambara, Thato Serite, Mike Sathekge, Mankgopo Kgatle

**Affiliations:** ^1^ Department of Nuclear Medicine, University of Pretoria and Steve Biko Academic Hospital, Pretoria, South Africa; ^2^ Nuclear Medicine and Research Infrastructure (NuMeRI), Department of Basic and Translational Research, Pretoria, South Africa; ^3^ Department of Biomedical Sciences, Tropical Diseases Research Centre (TDRC), Ndola, Zambia; ^4^ Department of Medicine, University of Cape Town and Groote Schuur Hospital, Observatory, Cape Town, South Africa

**Keywords:** AHR, Nrf2, HIF-1α, PPARγ, NF-κB, IRF, ATF3, STAT

## Abstract

SARS-CoV-2, the virus responsible for COVID-19, interferes with the host’s transcriptional control systems, triggering widespread disruption of immune regulation and metabolic stability. Key transcription factors (TFs), including AHR, NRF2, NF-κB, IRFs, HIF-1α, PARP, STAT3, ATF3, and PPARγ, play crucial roles in inflammation, oxidative stress defence, anti-viral responses, and immunometabolic adaptation. Their activity and interactions are modulated by post-translational modifications (PTMs) such as phosphorylation, SUMOylation, and ubiquitination, which shape COVID-19 progression. Specifically, SUMOylation of PPARγ suppresses NF-κB-driven inflammation, though impairment under severe disease amplifies macrophage activation and cytokine release. NRF2 degradation via KEAP1–CUL3–mediated ubiquitination is manipulated by the virus to deregulate oxidative stress responses, while SARS-CoV-2 also modulates NF-κB activity through ubiquitination of viral proteins (e.g., NSP6, ORF7a). Dynamic crosstalk between AHR and NRF2 further illustrates TF duality in detoxification and inflammation, with SUMOylation potentially influencing nuclear retention and transcriptional precision. This review classifies transcription factors into four functional categories: inflammatory regulators, antiviral response mediators, stress and pathogen response elements, and metabolic modulators. It further examines how PTM–driven crosstalk contributes to immune dysregulation. Targeting these transcriptional networks presents promising therapeutic strategies to mitigate hyperinflammation, rebalance immune responses, and enhance clinical outcomes in COVID-19.

## Introduction

Severe acute respiratory syndrome coronavirus 2 (SARS-CoV-2) is a positive-sense RNA virus responsible for causing coronavirus disease 2019 (COVID-19) (Lai et al., 2020). The interaction between COVID-19 and host transcription factors (TFs) is complex and multifaceted (Santoni et al., 2023). SARS-CoV-2 not only infects host cells but also manipulates their transcriptional machinery, leading to dysregulated immune responses and enhanced viral replication (Zhang et al., 2024). This disruption can exacerbate disease severity by interfering with essential cellular processes such as transcription.

TFs are pivotal regulators of gene expression, orchestrating immune and metabolic responses to external stimuli, including viral infection. During SARS-CoV-2 infection, the virus disrupts host transcriptional machinery, altering TF activity to subvert immune defences and reshape cellular metabolism (Santoni et al., 2023; Liu et al., 2024; Zhang et al., 2024; Patalano et al., 2025). This manipulation contributes to immune dysregulation, inflammatory cascades, and tissue damage observed in severe COVID-19 cases. Several key TFs, such as aryl hydrocarbon receptor (AHR), nuclear factor erythroid 2-related factor 2 (NRF2), nuclear factor kappa-light-chain-enhancer of activated B cells (NF-κB), interferon regulatory factors (IRFs), hypoxia-inducible factor-1α (HIF-1α), peroxisome proliferator-activated receptor gamma (PPARγ), activating transcription factor 3 (ATF3), poly(ADP-ribose) polymerase (PARP), and signal transducer and activator of transcription 3 (STAT3), play crucial roles in regulating inflammation, oxidative stress, antiviral defence, and immunometabolic adaptation (Tian et al., 2021; Hamad et al., 2023; Hasankhani et al., 2023; Kesika et al., 2024; Liu et al., 2024; Xu et al., 2024; Patalano et al., 2025). Altered expression or activity of these TFs may exacerbate hyperinflammatory responses and contribute to immune cell depletion, especially of T and NK cell populations during the acute phase of infection (Frank and Paust, 2020; Sayahinouri et al., 2023; Yip et al., 2024). The coordinated regulation of these TFs, often governed by cytoplasmic retention and nuclear translocation in response to environmental triggers such as oxidative stress and hypoxia, is central to COVID-19 pathogenesis (Guryanova et al., 2025). Understanding their functional roles provides a framework for developing targeted therapies aimed at restoring immune balance.

To build this framework, the review systematically classifies TFs according to their activation mechanisms, subcellular localization, and functional roles in disease progression. These factors are grouped into four functional groups: inflammatory regulators, antiviral response mediators, stress and pathogen response elements, and metabolic modulators. The therapeutic relevance of each group is examined in detail, underscoring how strategic modulation of transcriptional networks may alleviate hyperinflammation, rebalance immune responses, and improve clinical outcomes in COVID-19.

## Host transcriptional regulation in respiratory infections

Host transcriptional regulation plays a crucial role in the immune response to respiratory infections by modulating gene expression profiles (Zhai et al., 2015). This process supports effective immune defence and is governed by various TFs and signalling pathways that respond to pathogen-associated molecular patterns (PAMPs) and damage-associated molecular patterns (DAMPs) (Ahmed-Hassan et al., 2020).

Respiratory infections caused by influenza viruses and SARS-CoV-2 influence the activation of the interferon (IFN) signalling pathway in host cells, leading to the upregulation of IFN-stimulated genes (ISGs) that enhance anti-viral defences (Wu and Metcalf, 2020; Znaidia et al., 2022). Studies have demonstrated that genes associated with the interferon pathway remain significantly upregulation during acute respiratory viral infections, persisting for several days post-infection (Zhai et al., 2015). For example, a study by Romel (Mackelprang et al., 2023) examined ISG expression in HIV-1 infection and revealed that genes linked to both type I and type II interferons (IFNs) were consistently or transiently upregulated, underscoring the synergistic interaction between these IFN types in ISG activity (Mackelprang et al., 2023). Similarly, Lee’s study (Lee et al., 2020) comparing immune responses to influenza and COVID-19 revealed that tumour necrosis factor-alpha (TNFα) and Interleukin-1 (IL-1) were dominant in COVID-19 cells, whereas ISGs were strongly upregulated in influenza infection (Lee et al., 2020).

Beyond viral infections, bacterial pathogens such as Streptococcus pneumoniae induce transcriptional changes in neutrophils that are essential for antimicrobial functions (Bhalla et al., 2021). Additionally, aging has been shown to impact neutrophils’ transcriptional response, impairing bacterial clearance and altering immune efficiency (Van Avondt et al., 2023). Age-specific transcriptional regulation also plays a role in respiratory infections. For example, children exhibit a more robust and earlier induction of interferon-related pathways following SARS-CoV-2 compared to adults, potentially contributing to milder disease outcomes in paediatric populations (Chen et al., 2020; Woodall et al., 2024).

Disruptions in transcriptional regulation can lead to inappropriate immune responses, increasing susceptibility to severe viral infections (Liu X. et al., 2020). An excessive immune response may result in cytokine storms, which can have life-threatening consequences, particularly in severe COVID-19 cases (Hu et al., 2021). A deeper understanding of host transcriptional regulation during respiratory infections is critical for advancing targeted therapeutic strategies. By elucidating the molecular mechanisms underlying these responses, we can identify potential interventions that strengthen immune defences without relying solely on pharmacological treatments.

## Transcription factor activation in COVID-19

The above-listed key TFs serve as key regulators of immune and inflammatory gene expression during SARS-CoV-2 infection. These TFs are typically sequestered in the cytoplasm in inactive states by molecular chaperones or inhibitory partners. Upon stimulation, through ligand binding, phosphorylation, oxidative stress, or other post-translational modifications, they translocate to the nucleus and initiate transcriptional programs that modulate cytokine production, antiviral responses, and immune cell behaviour (Liu et al., 2016).

Among the TFs discussed, NF-κB, STAT1, STAT3, IRF1/3/7, and ATF3 act as principal upstream regulators of TNF-α and IFN-γ, which are central mediators of cytokine storm syndromes in severe COVID-19. NF-κB is the dominant regulator of TNF-α expression, activated by inflammatory stimuli such as TNF-α itself, IL-1β, and viral components via IKK-dependent degradation of its inhibitor IκB (Oeckinghaus and Ghosh, 2009; Hadjadj et al., 2020). STAT1 and IRF1 are induced by IFN-γ transcription following interferon and cytokine signalling (Qiao et al., 2013), while STAT3 interacts with NF-κB at gene promoters like fascin, as demonstrated by Snyder et al (Snyder et al., 2014), where ChIP assays revealed transcriptional synergy in response to IL-6 and TNF-α (Snyder et al., 2014). ATF3, activated through the integrated stress response, functions as a fine-tuner and repressor of pro-inflammatory genes including TNF-α, and helps constrain excessive immune activation (Schmitz et al., 2018; Badu et al., 2024).

The dysregulation of these TFs, whether through excessive activation, impaired negative feedback, or delayed induction, can lead to uncontrolled cytokine release and escalate hyperinflammatory pathology. For example, delayed type I IFN responses, as reported in severe COVID-19 cases, compromise early antiviral defences and intensify downstream inflammation (Song et al., 2020). Impaired IRF7 function further amplifies IFN-I signalling and disrupts immune regulation (Ma et al., 2023). Persistent NF-κB activation sustains TNF-α transcription and inflammation, while IFN-γ production leads to aberrant STAT1/STAT3 signalling drives and immune cell recruitment, contributing to tissue injury and disease severity (Tak and Firestein, 2001; Yu et al., 2017).

Adding to this regulatory complexity, HIF-1α, NRF2, and PPARγ play modulatory roles that intersect with both inflammatory and antiviral pathways. HIF-1α, stabilized under hypoxic conditions common in COVID-19-associated lung injury, promotes the expression of genes that support glycolytic metabolism and immune activation. It’s cooperative interaction with NF-κB can enhance transcription of TNF-α and IL-1β, facilitating macrophage polarization toward a pro-inflammatory state (Wang et al., 2014). In contrast, NRF2 responds to oxidative stress by escaping Keap1-mediated degradation and activating cytoprotective genes. Through repression of NF-κB signalling and reduction of oxidative damage, NRF2 dampens TNF-α and IFN-γ expression, providing a counterbalance within the transcriptional network (Noel et al., 2015; Kobayashi et al., 2016; Ngo and Duennwald, 2022). PPARγ, upon ligand activation, antagonizes NF-κB and STAT pathways, suppresses TNF-α transcription, and promotes immune resolution via macrophage deactivation and metabolic reprogramming (Harmon et al., 2011; Croasdell et al., 2015). Deficiency or downregulation of NRF2 and PPARγ may weaken these regulatory checkpoints, leaving inflammatory cascades unrestrained.

Crosstalk and feedback loops further entrench this network. STAT1 and NF-κB, for instance, can be co-activated by TLR ligands and IFNs, resulting in joint promoter occupancy and enhanced cytokine transcription (Hu and Ivashkiv, 2009; Piaszyk-Borychowska et al., 2019). NF-κB indirectly boosts IFN-γ expression via IL-12/IL-18 stimulation, forming a positive feedback loop (Kannan et al., 2011). Meanwhile, ATF3’s dual functionality, which includes simultaneously suppressing viral replication and regulating inflammatory gene expression, positions it as a central modulator that balances the immune system’s pro and anti-inflammatory axes (Badu et al., 2024; Liu et al., 2024).

Altogether, this dynamic and overlapping transcriptional architecture that governs the expression of TNF-α, IFN-γ, and IFN-I operates under tight temporal and spatial control. Dysregulation at multiple regulatory nodes can disrupt homeostatic mechanisms and trigger cytokine storm syndromes. Evidence from (Karki et al., 2020) underscores that combined inhibition of TNF-α and IFN-γ, rather than targeting either cytokine alone, is required to effectively mitigate cytokine storm and rescue lethally infected mice (Karki et al., 2020). These findings emphasize the importance of targeting multiple transcriptional regulators and signalling pathways concurrently to achieve meaningful therapeutic outcomes.

In conclusion, clarifying the hierarchical organization and cooperative interactions among NF-κB, STATs, IRFs, ATF3, HIF-1α, NRF2, and PPARγ significantly enhances our understanding of immune dysregulation in COVID-19. This integrated knowledge provides a foundation for developing combinatorial transcription factor–based strategies that aim to reduce hyperinflammation while preserving essential antiviral responses. Such approaches offer a rational and clinically relevant framework to improve patient outcomes across diverse disease presentations.

## Inflammatory drivers in COVID-19

### Pro-inflammatory drivers

AHR, NF-κB, and STAT3 drive pro-inflammatory responses during viral infections, particularly SARS-CoV-2. Their dysregulation contributes to excessive inflammation, cytokine storm syndrome, and immune exhaustion, all of which are characteristic of severe COVID-19.

NF-κB plays a central role in immune signalling by activating the transcription of pro-inflammatory cytokines such as IL-6, TNF-α, and IL-1β (**Table 1**) (Liu et al., 2017). SARS-CoV-2 can stimulate NF-κB via angiotensin-converting enzyme 2 (ACE2), leading to uncontrolled inflammation, immune suppression, and tissue damage, particularly in severe cases (Zhou et al., 2024). Overactivation of NF-κB has been directly linked to cytokine storm syndrome due to excessive transcription of pro-inflammatory cytokines and chemokines, accelerating disease progression and worsening outcomes (Majidpoor and Mortezaee, 2022; Guo et al., 2024).

**Table 1:** Classification of key TFs based on function, regulation, and COVID-19 implications.

TFs	Location	Function	Ligand types	Relevant PTMs	Effects on TF localisation	COVID-19 implication
AHR	Cytoplasmic→ Nuclear	Immune modulation	Environmental toxins (e.g., dioxins), tryptophan metabolites	Ubiquitination (Farooqi et al., 2023)	Promotes nuclear translocation upon ligand activation (Farooqi et al., 2023).	Drives cytokine storm; activates ACE2 (Hord et al., 2000; Liu et al., 2017; Guarnieri, 2022; Xu et al., 2024)
NF-κB	Cytoplasmic→ Nuclear	Pro-inflammatory gene expression	Pro-inflammatory cytokines, TLR ligands	Phosphorylation SUMOylation & viral protein ubiquitination (Nishitsuji et al., 2022)	Triggers pro-inflammatory cytokine expression (Hariharan et al., 2021). Amplify NF-κB-driven inflammation (Hariharan et al., 2021).	Central in cytokine storm, T cell exhaustion (Oeckinghaus and Ghosh, 2009; Liu et al., 2017; Gudowska-Sawczuk and Mroczko, 2022)
STATs	Cytoplasmic→ Nuclear	IFN signalling, immune response	Cytokines (e.g., IL-6, IFNs)	Phosphorylation (Znaidia et al., 2022).	Enables nuclear translocation and transcriptional activation (Znaidia et al., 2022).	STAT1 downregulated, STAT3 drives fibrosis (Jafarzadeh et al., 2021; Vázquez-Jiménez et al., 2021)
PPARγ	Nuclear	Anti-inflammation	Fatty acids, thiazolidinediones	SUMOylation (Ghisletti et al., 2007).	Suppresses inflammatory signalling (Ghisletti et al., 2007).	Downregulated; ligand activation shows therapeutic promise (Hasankhani et al., 2023)
NRF2	Cytoplasmic→ Nuclear	Anti-inflammation	Electrophiles, oxidants	Inhibited ubiquitination (due to SARS-CoV-2 oxidative stress) (Kombe Kombe et al., 2024).	Stabilizes NRF2 and enhances nuclear localization and transcriptional activity (Theodore et al., 2008).	Suppressed in COVID-19; protective against oxidative damage (Muchtaridi et al., 2022; Wang et al., 2023)
ATF3	Nuclear	Anti-inflammation	Oxidative stress, ER stress, cytokines (e.g., IL-6, TNF-α), lipopolysaccharide (LPS), and metabolic intermediates (e.g., erastin, naringin)	- SUMOylation and ubiquitination (Li et al., 2023a).	ATF3 is stable under normal conditions and the expression increases during inflammation (Li et al., 2023a).	Regulates inflammation; protective role suspected (Sun and Chang, 2022; Li et al., 2023b; Badu et al., 2024; Liu et al., 2024)
IRFs (IRF3/7)	Cytoplasmic/Nuclear	IFN production, antiviral defence	Viral nucleic acids via TLR/RIG-I pathways	Phosphorylation (Lin et al., 1998).	Regulates IFN-α/β production, supporting antiviral responses (Chen et al., 2013).	Suppressed by virus; vital for antiviral immunity (Chiang and Liu, 2018; Wu and Metcalf, 2020; Ma et al., 2023)
HIF-1α	Cytoplasmic→ Nuclear	Hypoxia response, inflammation	Hypoxia, succinate, nitric oxide, growth factors, inflammatory cytokines (e.g., TNF-α), and certain fibrates (e.g., bezafibrate	–	No known PTM relevance reported	Activates pro-inflammatory cytokines, worsens lung injury (Liu et al., 2020b)

AHR, traditionally recognized for its role in environmental toxin responses, has emerged as a significant regulator of inflammation in COVID-19 (**Table 1**). The virus manipulates AHR signalling to suppress antiviral defences while amplifying the production of IL-1, IL-6, and IL-18, contributing to neutrophil recruitment and increased ROS generation, factors that further intensify inflammation (Mittal et al., 2014; Anderson et al., 2020; Ren et al., 2020). AHR also modulates cytokine expression, including IFN-γ and TNF-α, influencing immune function through its regulation of indoleamine 2,3-dioxygenase 1 (IDO-1). IFN-γ stimulates IDO-1, producing kynurenine, a metabolite required for AHR activation (Xu et al., 2024). Beyond direct cytokine regulation, AHR interacts with multiple inflammatory pathways, including NF-κB, STAT3, epidermal growth factor receptor (EGFR), and HIF, reinforcing immune dysregulation (Xu et al., 2024).

STAT3, a key player in the JAK/STAT signalling pathway, primarily drives inflammatory and fibrotic responses (**Table 1**). Although STAT1 and STAT2 play protective roles by supporting interferon-mediated antiviral responses, aberrant activation of STAT3 has been linked to lung fibrosis and immune dysregulation in both acute and prolonged phases of COVID-19 (Hahm et al., 2005; Jafarzadeh et al., 2021). This dysregulation amplifies IL-6 signalling, perpetuates inflammation, and promotes excessive cytokine production, contributing to immune cell infiltration and tissue injury (Majidpoor and Mortezaee, 2022). In its inactive state, STAT3 remains in the cytoplasm until IL-6 binds to its receptor (IL-6Rα), triggering activation. A wide range of inflammatory cytokines associated with COVID-19, including TNF-α, IFN-γ, IL-5, IL-9, IL-10, IL-11, granulocyte colony-stimulating factor (G-CSF), macrophage colony-stimulating factor (M-CSF), monocyte chemotactic protein-1 (MCP-1), and chemokine (C-C motif) ligand 5 (CCL5), can activate STAT3, further exacerbating inflammation (Jafarzadeh et al., 2021).

Dysregulated STAT3 activation, combined with suppressed type I interferon responses and excessive IL-6 and TNF-α production, weakens viral containment, facilitating the spread of SARS-CoV-2 and contributing to systemic COVID-19 symptoms (Matsuyama et al., 2020; Eskandarian Boroujeni et al., 2022). IL-6 utilizes two distinct Janus kinases (JAK)/STAT signalling pathways: classical cis-signalling, where IL-6 binds membrane-bound IL-6R (mIL-6R) to form a complex with gp130 that activates JAK/STAT3; and trans-signalling, in which IL-6 binds soluble IL-6R (sIL-6R), forming an IL-6/sIL-6R/gp130 complex that amplifies inflammatory mediators such as VEGF and MCP-1. Excessive IL-6 production through trans-signalling is a major driver of cytokine storms in severe COVID-19 (Jiang et al., 2022).

Together, NF-κB, AHR, and STAT3 contribute to immune dysregulation in COVID-19 by sustaining inflammation, promoting cytokine storm syndrome, and exacerbating tissue damage. Their involvement in disease progression underscores their significance as potential therapeutic targets for controlling severe COVID-19.

### Anti-inflammatory and immune-modulatory roles

During SARS-CoV-2 infection, AHR, NRF2, PPARγ, and ATF3 play essential roles in regulating immune responses, controlling inflammation, and maintaining immune balance. Their activation or suppression significantly influences disease progression, either mitigating or exacerbating inflammatory damage.

AHR is an immunomodulatory TF activated via both IDO1-dependent and independent pathways, shaping cytokine expression (including IL-1β, IL-10, and TNF-α) and influencing immune signalling, as described in **Table 1** (Turski et al., 2020; Xu et al., 2024). By suppressing NF-κB and type I IFN pathways, dysregulated AHR helps control hyper-inflammation and reduces the severity of cytokine storms (Øvrevik et al., 2014; Xu et al., 2024). Additionally, AHR has been implicated in regulating ACE2 expression, potentially influencing viral entry into host cells (Guarnieri, 2022).

NRF2, a master regulator of oxidative stress, plays a critical role in dampening inflammation by reducing reactive oxygen species (ROS), inhibiting NF-κB, and modulating type I IFN signalling via the STING pathway (Park et al., 2023; Wang et al., 2023; Zhang et al., 2023). However, SARS-CoV-2 disrupts NRF2 function by activating PKR, leading to NRF2 degradation, increased ROS levels, endothelial dysfunction, and impaired tissue repair, key factors that contribute to cytokine storm development (Hamad et al., 2023). Clinical studies highlight reduced NRF2 levels in COVID-19 patients, particularly in children, reinforcing its protective role in mitigating disease severity (Gümüş et al., 2022).

PPARγ, widely recognized for its anti-inflammatory function, promotes M2 macrophage polarization, which reduces immune activation and inflammation (**Table 1**) (Feng et al., 2016). By inhibiting NF-κB and STAT pathways, PPARγ suppresses pro-inflammatory cytokines such as TNF-α, IL-6, IL-1β, and MCP-1, minimizing lung damage and tissue deterioration (Hasankhani et al., 2023; Kim et al., 2023). PPARγ also interacts with RXR to further suppress inflammation (Plutzky and Kelly, 2011). A decline in PPARγ levels during SARS-CoV-2 infection correlates with heightened inflammatory responses, contributing to severe disease outcomes (Hasankhani et al., 2023).

Collectively, AHR, NRF2, PPARγ, and ATF3 maintain immune homeostasis by limiting inflammation and preventing uncontrolled immune activation. Their dysregulation contributes to COVID-19 severity, highlighting their potential as therapeutic targets for immune modulation strategies.

## Interferon regulation and antiviral defence

IFN signalling is a critical component of the innate immune defense against viral infections, orchestrated by key transcription factors such as IRFs, STATs, and ATF3. When SARS-CoV-2 enters host cells, PRRs such as retinoic acid inducible gene-I (RIG-I), melanoma differentiation-associated protein 5 (MDA5), and toll-like receptors (TLRs) detect viral RNA, triggering immune signalling cascades that activate IRF3, IRF7, and NF-κB. This activation leads to the production of type I and III IFNs, which bind to their respective receptors and initiate the JAK-STAT pathway (**Table 1**). The downstream effect of this activation is the induction of ISGs, which suppress viral replication, degrade viral RNA, and enhance antigen presentation (Ma et al., 2023; Minkoff and tenOever, 2023; Qing and Liu, 2023).

SARS-CoV-2 causes a dysregulated IFN response, overproduction of inflammatory cytokines in the lungs (Eskandarian Boroujeni et al., 2022). This impaired IFN signalling also leads to immune cell infiltration that elevates lung cytokines and ultimately vascular damage (Eskandarian Boroujeni et al., 2022). However, SARS-CoV-2 has developed strategies to evade IFN responses, particularly during early infection. Viral proteins such as NSP1, NSP6, NSP13, and ORF6 disrupt IRF3 phosphorylation and hinder the nuclear import of STAT1/STAT2, effectively reducing ISG activation and allowing viral replication to persist unchecked (Samuel, 2023). In severe cases of COVID-19, especially among older individuals and those with comorbidities, type I IFN production (IFN-α and IFN-β) is markedly diminished, contributing to excessive inflammatory responses and cytokine storms that exacerbate disease progression (Pietrobon et al., 2020).

STAT proteins are crucial to IFN-mediated antiviral responses, with STAT1 and STAT2 forming the interferon-stimulated gene factor 3 (ISGF3) complex to regulate ISG expression (**Table 1**) (Platanitis and Decker, 2018). While these proteins are central to immune defence, STAT3 dysregulation is linked to chronic inflammation and fibrosis, complicating recovery from COVID-19 (Kasembeli et al., 2018). Additionally, ATF3 plays a dual regulatory role in antiviral defence and immune modulation by enhancing STAT1-mediated ISG expression while simultaneously tempering excessive inflammation through NF-κB inhibition as shown in **Table 1** (Badu et al., 2024; Liu et al., 2024). Persistent activation of STAT1 increases IFN-γ production and ISG overload (Zhang et al., 2006). ATF3 helps prevent immunopathology by controlling the expression of key cytokines, including TNF-α, IL-6, and CCL2 (Liu et al., 2024). However, its dysregulation can result in either exaggerated inflammation or impaired antiviral responses, both of which are defining features of severe COVID-19 (Labzin et al., 2015).

The pathogenesis of SARS-CoV-2 involves intricate disruptions in IFN signalling, with impaired IRF, STAT, and ATF3 function weakening antiviral defences and promoting immune dysregulation. These disruptions contribute significantly to severe disease progression, making these pathways potential targets for therapeutic intervention.

## Cellular response mechanisms in COVID-19

### Response to stress and pathogens

SARS-CoV-2 infection induces severe oxidative stress, hypoxia, and immune dysregulation, triggering ATF3, NRF2, HIF-1α, and STAT proteins. These regulators coordinate responses to inflammation, apoptosis, and ferroptosis, an iron-dependent form of cell death associated with viral stress.

NRF2 plays a central role in counteracting oxidative damage by activating genes involved in cellular detoxification (**Table 1**) (Ngo and Duennwald, 2022). However, SARS-CoV-2 can manipulate NRF2 activity, either enhancing it to facilitate viral replication or suppressing antioxidant defences, worsening oxidative injury (Qu et al., 2023). The virus exploits the phosphatidylinositol 3-kinase (PI3K)/Akt/NRF2 pathway to modulate cellular survival and intracellular trafficking (Lekshmi et al., 2023), while excessive reactive oxygen species (ROS) accumulation can trigger ferroptosis, leading to tissue damage (Liu et al., 2023). SARS-CoV-2 open reading frame 3a (ORF3a) is known to regulate ferroptosis via the Keap1-NRF2 axis, influencing oxidative stress response (Liu et al., 2023). By modulating genes like glutathione peroxidase 4 (GPX4), NRF2 alters cellular responses to stress, ultimately inducing ferroptosis (Kombe Kombe et al., 2024). Additionally, SARS-CoV-2 ORF6 disrupts intracellular redox balance by antagonizing NRF2-mediated antioxidant pathways, exacerbating oxidative damage through p38 activation (De Angelis et al., 2023).

ATF3, a stress-responsive transcription factor, interacts with NRF2 to enhance cytoprotective mechanisms. While typically stable, ATF3 expression is upregulated under oxidative and inflammatory stress (**Table 1**) (Sood et al., 2017). ATF3 can either inhibit or promote viral replication, depending on the pathogen (Badu et al., 2024). Additionally, it plays a significant role in apoptosis and ferroptosis, influencing cell survival and tissue integrity in response to viral-induced stress (Liu et al., 2024).

HIF-1α, a key regulator of hypoxia adaptation, stabilizes under low-oxygen conditions and modulates inflammation via vascular endothelial growth factor (VEGF), erythropoietin (EPO), and heme oxygenase-1 (HO-1) (Ramakrishnan et al., 2014; Cheng et al., 2017). However, excessive HIF-1α activation contributes to cytokine storm syndrome, worsening lung injury, and this is shown in **Table 1** (Jahani et al., 2020). SARS-CoV-2 induces mitochondrial dysfunction, inhibiting oxidative phosphorylation (OXPHOS) while enhancing mitochondrial ROS production. This leads to the release of mitochondrial DNA (mtDNA), triggering immune activation (Guarnieri et al., 2024). The viral ORF3a protein further amplifies HIF-1α expression, driving inflammation and viral pathogenesis (Tian et al., 2021). Hypoxia can be exacerbated by PAMPs and DAMPs, reinforcing HIF-1α activation in immune cells (Serebrovska et al., 2020). Interestingly, HIF-1α may exert cardioprotective effects, as seen in COVID-19 patients, where it preserved endothelial integrity and reduced cell death (Wang BJ. et al., 2021).

The JAK/STAT pathway is central to cytokine signalling, immune regulation, and inflammation (Mahjoor et al., 2023). In COVID-19, dysregulated JAK/STAT activation contributes to immune hyperactivation and cytokine storm syndrome, worsening disease severity (Rodriguez and Carnevale, 2024). SARS-CoV-2 selectively suppresses STAT1-mediated interferon responses while overactivating STAT3, promoting inflammation and fibrosis (Chen et al., 2021).

Additionally, JAK/STAT activation influences host susceptibility in cases of SARS-CoV-2 and influenza co-infection. IFN-γ activates STAT1 and STAT2 through phosphorylation, but SARS-CoV-2’s non-structural protein 1 (nsp1) protein inhibits ISG expression, enabling immune evasion (Baral et al., 2024). Therapeutic interventions targeting this pathway, such as JAK/STAT inhibitors like baricitinib, have shown potential in mitigating hyper-inflammatory responses in severe COVID-19 (Zhang X. et al., 2022).

Together, ATF3, NRF2, HIF-1α, and STAT proteins regulate cellular responses to SARS-CoV-2-induced stress, shaping immune adaptation and inflammation. Their dysregulation contributes to disease progression, making them promising targets for therapeutic intervention.

### Metabolic and cellular stress modulators

TFs such as HIF-1α and PPARγ are key modulators of metabolic homeostasis and immune responses during viral infections. Their dysregulation during SARS-CoV-2 infection has been implicated in disease progression through effects on inflammation, viral replication, and cellular stress responses.

Beyond its role in oxygen sensing, HIF-1α modulates immune cell function by promoting pro-inflammatory cytokine production (e.g., IL-1β, TNF-α), altering cellular metabolism toward glycolysis, and enhancing leukocyte recruitment (Palazon et al., 2014). The interplay between HIF-1α and PPARγ is particularly relevant in COVID-19, where immune dysregulation, metabolic reprogramming, and oxidative stress are hallmarks of severe disease (**Table 1**). While HIF-1α promotes a glycolytic, pro-inflammatory state conducive to viral replication, PPARγ counteracts this by fostering metabolic balance and dampening immune overactivation (de Carvalho et al., 2021; Shen and Wang, 2021; Rudiansyah et al., 2022). Consequently, targeting this axis therapeutically holds promise for mitigating inflammation and improving disease outcomes in COVID-19 and other viral infections.

SARS-CoV-2 exploits this metabolic pathway to enhance viral replication and inflammatory responses. The ORF3a protein upregulates HIF-1α expression, creating a hyper-inflammatory environment that facilitates viral survival (Tian et al., 2021). Furthermore, SARS-CoV-2-induced mitochondrial ROS stabilise HIF-1α, driving the glycolytic switch in monocytes and macrophages and amplifying cytokine production (Shouman et al., 2024). Similar mechanisms have been observed in respiratory syncytial virus (RSV) and hepatitis B virus (HBV), where HIF-1α stabilisation promotes cell survival, suppresses apoptosis, and weakens antiviral signalling (Huang et al., 2021; Riedl et al., 2021).

In contrast, PPARγ serves as a key counter-regulatory transcription factor that maintains metabolic equilibrium and restricts excessive immune activation. Though primarily recognized for its role in glucose and lipid metabolism, PPARγ also exerts anti-inflammatory effects by inhibiting NF-κB and STAT signalling pathways (Chi et al., 2021; Moon et al., 2024). Its activation leads to reduced expression of pro-inflammatory cytokines such as IL-6, TNF-α, and IL-1β while promoting the M2 anti-inflammatory macrophage phenotype and facilitating tissue repair (Yu et al., 2023).

PPARγ further mitigates HIF-1α-driven inflammation by inducing arginase-1, an enzyme associated with immune resolution and tissue healing (Yu et al., 2023). Experimental studies have demonstrated that PPARγ-deficient macrophages exhibit elevated pro-inflammatory cytokine production and impaired resolution of inflammation following immune challenges (Yu et al., 2023).

## Potential crosstalk and clinical relevance in COVID-19

The regulation and crosstalk among the key TFs may play pivotal roles in the progression of COVID-19 from mild, moderate to severe disease. These factors are modulated through shared signalling pathways and various post-translational modifications, influencing the host’s immune response and disease outcome.

## Signalling pathway-mediated crosstalk in COVID-19

### JAK/STAT and IRFs signalling in interferon response

**Figure 1:**
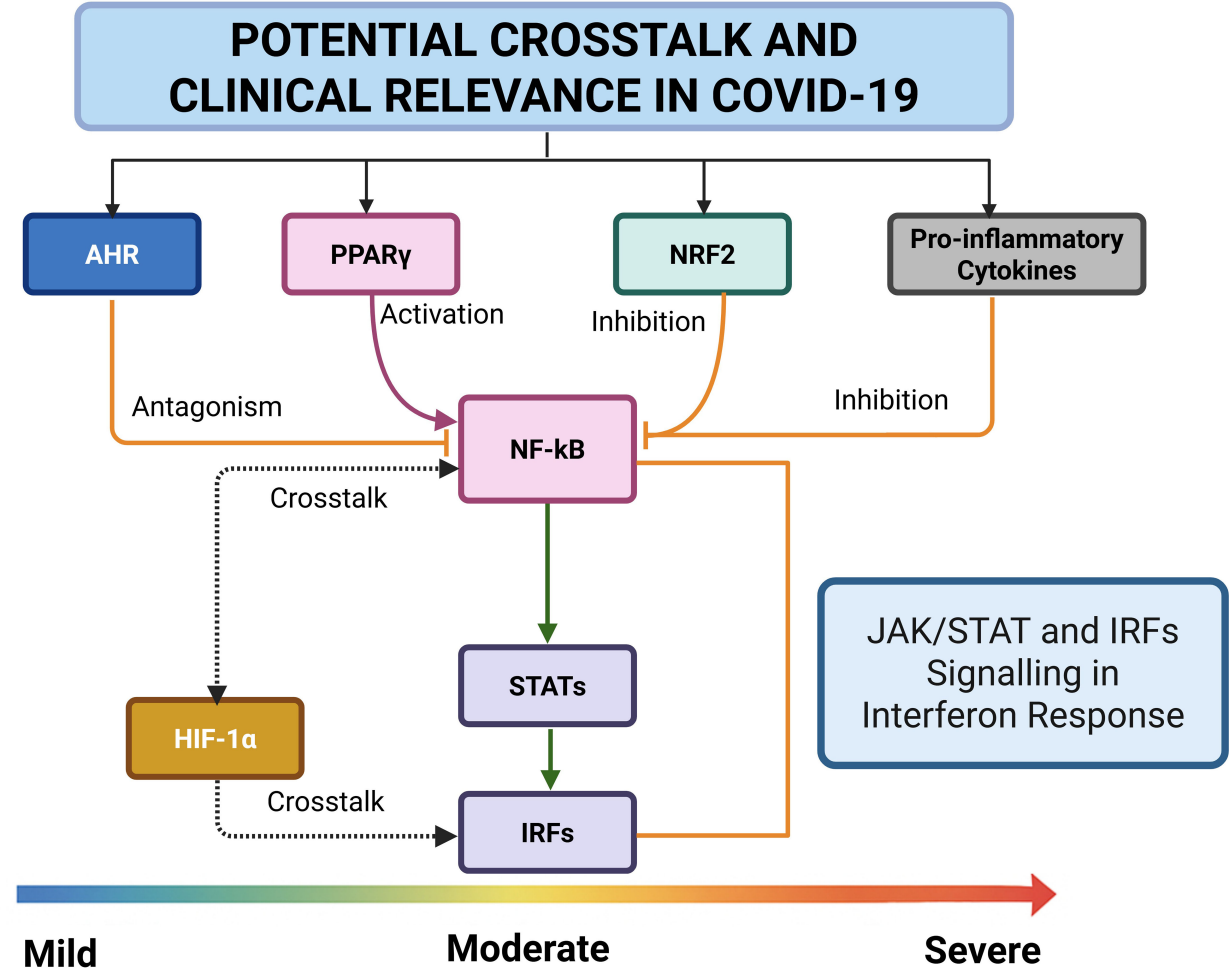
Illustration of the subcellular interactions of key TFs in immune and stress responses. AHR is typically located in the cytoplasm, bound to a complex. Upon activation, it translocates into the nucleus, where it competes with HIF-1α for ARNT binding, and bind to XRE, influencing gene transcription. NRF2, typically inhibited by KEAP1, becomes activated under oxidative stress and interacts with NF-κB, which also induces ATF3 to support STAT activation. STATs promote IFN signalling, counteracting NF-κB pathways. Proinflammatory cytokines drive NF-κB activation via the IKK complex, leading to STAT phosphorylation, nuclear translocation, and IRF expression, further enhancing IFN activation.

The JAK/STAT and IRF signalling pathways are integral to the IFN response, serving as key regulators of antiviral immunity (**Figure 1**). Upon cytokine stimulation, the JAK/STAT pathway initiates a cascade wherein JAKs phosphorylate their associated receptors, allowing STAT proteins to dock, undergo phosphorylation, and form dimers. These activated STAT dimers then translocate to the nucleus, where they bind to specific DNA sequences, triggering the transcription of ISGs that enhance the anti-viral response (Morris et al., 2018; Yan et al., 2018; Dai et al., 2022).

IRFs, particularly IRF3 and IRF7, are also activated in this pathway, amplifying IFN production and strengthening immune defences. DNA sensors such as cyclic GMP-AMP synthase (cGAS) detect viral infections and activate the stimulator of IFN genes (STING) adaptor, which then translocates to the Golgi. Here, STING phosphorylation facilitates the recruitment of IRF3, which subsequently migrates to the nucleus to induce IFNs and ISGs (Lukhele et al., 2019). These DNA sensors are themselves ISGs, meaning they are upregulated in response to viral infections, reinforcing IFN production and enhancing the overall immune response (Lukhele et al., 2019).

The activation of IRF3 and IRF7 leads to the induction of IFN-stimulated response elements (ISRE) and IRF-binding elements (IRFE), further modulating ISG expression through the JAK/STAT pathway (Ning et al., 2005). IRF7 plays a pivotal role in IFN-β production through its interaction with STING and IRF3 at the promoter site. Meanwhile, IRF1 regulates ISG expression and enhances IRF9 activity, which, in turn, supports IRF7 through the interferon-stimulated gene factor 3 (ISGF3) complex. Additionally, several IRF and STAT genes rely on serum- and glucocorticoid-regulated kinase 3 (SGK3), while IRF4 uniquely depends on SGK1 for its regulatory functions (Castillo Cabrera et al., 2023; Ma et al., 2023).

Importantly, IRF7 governs the regulation of IFN-I genes downstream of PRRs, forming a positive feedback loop that sustains IFN production to combat viral infections effectively (Qing and Liu, 2023). Disruptions in this pathway, as observed in severe cases of SARS-CoV-2 infection, can hinder IFN responses, reduce antiviral defence and exacerbe disease progression.

### NF-κB, STAT3, and HIF-1α signalling in inflammation

**Figure 2:**
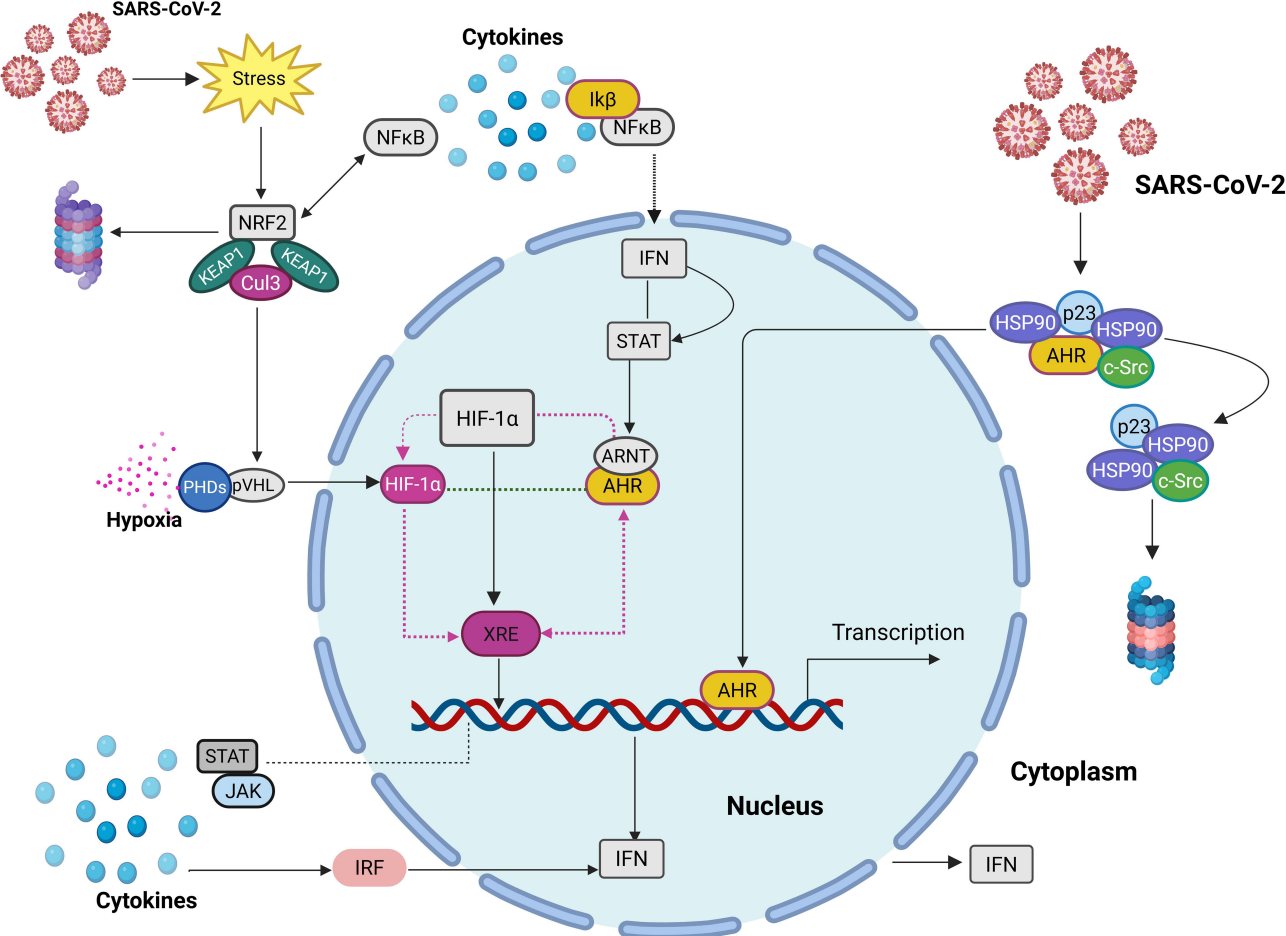
NF-κB acts as a central inflammatory mediator, modulated by upstream regulators such as AHR, PPARγ, NRF2, and disrupted IFN signalling. AHR can exert an antagonistic influence on NF-κB, dampening excessive inflammatory responses. The interplay between HIF-1α and NF-κB is particularly prominent under inflammatory hypoxia, where they mutually reinforce each other, exacerbating tissue injury. Normally, IκBα, the inhibitor of NF-κB, exerts a form of “inhibition” by sequestering NF-κB in the cytoplasm, thus preventing unwarranted activation. When this regulatory intoxication is lost, through IκBα degradation, NF-κB translocates to the nucleus, amplifying inflammation. NF-κB signalling further activates STATs and induces IRFs, modulating antiviral responses. Robust IRF activity promotes effective viral clearance and mild disease. In contrast, sustained NF-κB activation, unopposed by IκBα or AHR antagonism, drives cytokine storms, tissue hypoxia, and progression to moderate or severe COVID-19.

NF-κB, STAT3, and HIF-1α interact to regulate inflammation in COVID-19 as described in **Figure 1**. Their intricate signalling crosstalk plays a pivotal role in immune dysregulation, contributing to excessive inflammation, cytokine storm syndrome, and tissue damage in severe cases. NF-κB serves as a central regulator of immune responses, driving the transcription of pro-inflammatory cytokines in reaction to stress, infection, and cellular damage (**Figure 2**) (Fan et al., 2013). STAT3, activated downstream of cytokine signalling, influences various cellular processes, including immune suppression, apoptosis, and tissue repair. Meanwhile, HIF-1α, which is typically stabilized under hypoxic conditions, intensifies inflammation by activating pro-inflammatory genes and modulating NF-κB activity (Fan et al., 2013; Biddlestone et al., 2015; D’Ignazio et al., 2017).

NF-κB and STAT3 are rapidly activated by inflammatory stimuli such as cytokines and cellular stress. Their activation triggers the expression of genes involved in proliferation, survival, and immune regulation, amplifying inflammatory responses (Grivennikov and Karin, 2010). Notably, STAT3 activation in immune cells contributes to the accumulation of regulatory T cells (Tregs), which exert an immunosuppressive function and can prolong inflammation in COVID-19 (Rébé et al., 2013; Wang H. et al., 2021). Additionally, NF-κB enhances STAT3 activation (**Figure 2**), while STAT3 reciprocally reinforces NF-κB signalling, forming a self-sustaining loop that amplifies immune responses and worsens inflammation (He and Karin, 2011). Research indicates that NF-κB can directly modulate STAT3 activity, while STAT3 reciprocally influences NF-κB activation, emphasizing their interdependent role in immune regulation (He and Karin, 2011).

HIF-1α, primarily responsible for hypoxia adaptation, is transcriptionally controlled by NF-κB, enabling activation under oxygen-independent conditions during infections (van Uden et al., 2008). Hypoxia and infection-induced stress enhance HIF-1α signalling through NF-κB activation, promoting immune cell differentiation and antibody production. Interestingly, bacterial infections have been found to simultaneously increase NF-κB and HIF-1α levels, reinforcing immune cell activation (D’Ignazio et al., 2016). However, under prolonged inflammatory conditions, HIF-1α can suppress NF-κB transcriptional activity, tempering its inflammatory effects. This interaction suggests a nuanced regulatory network where HIF-1α can both enhance and regulate NF-κB activity, depending on disease severity and immune demands (Ruan et al., 2024).

In relation to COVID-19, HIF-1α plays a dual role in immune regulation and inflammation (Tian et al., 2021). While its activation helps coordinate immune responses against viral infections, excessive signalling leads to immune-related complications, particularly by escalating inflammatory activity in macrophages (Jahani et al., 2020; Tang et al., 2023). The intricate interplay between NF-κB, STAT3, and HIF-1α creates a dynamic signalling network that shapes COVID-19’s inflammatory profile. Understanding these interactions can inform potential therapeutic strategies, including NF-κB and STAT3 inhibitors, which may help mitigate immune hyperactivation and reduce disease severity.

### AHR, PPARγ, and NRF2 signalling at the metabolism-immunity interface

AHR, PPARγ, and NRF2 regulate the delicate balance between metabolism and immune responses. Their interactions shape inflammation, oxidative stress defence, and lipid metabolism, influencing both immune modulation and disease progression.

AHR plays a crucial role in modulating immune responses to environmental toxins and regulating cell proliferation and metabolism. It exerts its effects either by directly binding to gene promoters or by interacting with and modifying other signalling pathways (Pot, 2012; Trikha and Lee, 2020). NRF2, a master regulator of oxidative stress, orchestrates the expression of genes involved in antioxidant defence and detoxification. In addition to its protective effects, NRF2 influences metabolic processes and inflammation, with its activity controlled by transcriptional and post-transcriptional mechanisms that maintain cellular homeostasis (He et al., 2020).

Research by Wakabayashi (Wakabayashi et al., 2010) highlights the regulatory role of AHR in lipid metabolism, demonstrating its suppression of triglyceride production and involvement in adipogenesis in AHR-deficient mice. Interestingly, AHR and NRF2 share overlapping roles during cellular differentiation, with NRF2 modulating adipogenesis via interactions with AHR (Wakabayashi et al., 2010).

PPARγ, recognized for its pivotal role in lipid metabolism, also exerts significant anti-inflammatory effects (Varga et al., 2011). Studies have identified PPARγ-regulated genes as part of the NRF2-controlled gene network, suggesting a direct connection between these two pathways. Notably, experimental findings in mice have shown that increased PPARγ levels correlate with decreased NRF2 expression, reinforcing their functional interplay (Lee, 2017).

Through their interconnected signalling mechanisms, AHR, PPARγ, and NRF2 collectively modulate metabolic and immune responses while counteracting NF-κB-driven inflammation as illustrated in both **Figures 1**, **2**. Understanding their crosstalk provides insights into potential therapeutic strategies for controlling immune dysregulation and mitigating disease severity in conditions such as COVID-19.

### NF-κB and NRF2-Keap1 signalling pathway

NF-κB and NRF2-Keap1 are pivotal signalling pathways that regulate the body’s response to oxidative stress and inflammation. These transcription factors exhibit a complex interplay, where reduced NRF2 activity enhances NF-κB signalling, leading to increased production of pro-inflammatory molecules. Conversely, NF-κB can modulate NRF2 target gene expression by influencing its transcriptional and functional activity.

NRF2 plays a dual role in viral infections, either promoting or inhibiting disease progression depending on the context. However, in most cases, NRF2 activation provides a protective mechanism for host cells, reducing oxidative damage and inflammation during viral infection (Herengt et al., 2021; Wang et al., 2023). Similarly, NF-κB is a key regulator of pro-inflammatory gene expression and controls the function of both innate and adaptive immune cells, contributing to immune activation and inflammatory responses (Liu et al., 2017).

NRF2 mitigates oxidative stress by inducing the expression of antioxidant enzymes such as HO-1, which inhibits TNF-α-driven transcription of adhesion molecules that promote inflammation. By increasing HO-1 levels, NRF2 can suppress NF-κB activation and limit cytokine release, thereby reducing inflammation (Bhandari et al., 2021). Upregulation of NRF2 enhances antioxidant gene expression while concurrently suppressing NF-κB activity and neutralizing free radicals. This protective mechanism extends to mitigating the effects of intoxication by reducing oxidative stress and inflammation (Gao et al., 2021). Additionally, NRF2 maintains cellular levels of IκBα, a key inhibitor of NF-κB, thereby preventing NF-κB-mediated gene transcription and further limiting the detrimental impact of intoxication on cellular function (**Figures 1**, **2**). However, NF-κB activation can inhibit NRF2 function by downregulating the transcription of antioxidant response elements (ARE), impairing oxidative defence mechanisms (Ngo and Duennwald, 2022; Casper, 2023).

In summary, NRF2 deficiency intensifies NF-κB activity, promoting excessive inflammation and potentially leading to cytokine storms (Gao et al., 2021). Meanwhile, NF-κB’s regulation of NRF2 transcription can have both stimulatory and inhibitory effects on the expression of NRF2 target genes, further influencing immune responses and disease progression (Wardyn et al., 2015). Understanding the crosstalk between these pathways provides valuable insight into potential therapeutic interventions for mitigating inflammation in conditions such as COVID-19.

### IFN, HIF-1α, and AHR signalling pathway

The interplay between IFNs, HIF-1α, and AHR plays a crucial role in the progression of COVID-19. Upon SARS-CoV-2 infection, IFN-β and IFN-γ are induced, triggering AHR activation via the indoleamine 2,3-dioxygenase (IDO)-kynurenine (Kyn) pathway (Guarnieri, 2022). Previous study demonstrated that the IFN response to SARS-CoV-2 infection activates AHR signalling in lung epithelial cells, leading to the upregulation of mucin gene expression and increased mucus production, an effect that can contribute to respiratory complications in severe cases (Liu et al., 2020a).

Additionally, research by Lee et al. (2021) revealed that IFN-γ disrupts basal glycolysis in human coronary artery endothelial cells (HCAECs) through the IDO-Kyn-AHR signalling pathway, leading to reduced HIF-1α activity. This suggests that IFN-γ-induced metabolic shifts may impair hypoxia responses in vascular tissues (Lee et al., 2021). Furthermore, AHR and HIF-1α compete for the aryl hydrocarbon receptor nuclear translocator (ARNT) to form their respective transcriptionally active complexes. Under hypoxic conditions, HIF-1α exhibits a stronger affinity for ARNT, potentially inhibiting AHR-ARNT complex formation and suppressing AHR-mediated gene expression (Zhang M. et al., 2022).

HIFs are essential regulators of immune adaptation to low oxygen environments and have been linked to various inflammatory conditions (McGettrick and O’Neill, 2020). Hypoxia-induced signalling can strongly influence both immune and non-immune cells, exacerbating inflammatory responses and driving disease progression (Cummins et al., 2016). The intricate crosstalk between IFN, HIF-1α, and AHR highlights potential therapeutic targets for modulating immune and metabolic pathways in COVID-19 treatment.

## PTMs-driven crosstalk between STATs, IRFs, and NF-κB in COVID-19

Post-translational modifications (PTMs) are essential for regulating protein function, stability, and molecular interactions within biological systems. These chemical modifications, which occur after protein synthesis, involve the addition or removal of specific groups on amino acid residues, playing a fundamental role in cellular signalling (Ramazi et al., 2020; Zhong et al., 2023). PTMs influence protein activity, intracellular localization, structural integrity, and molecular interactions, shaping both normal physiological functions and disease mechanisms. Key PTMs include phosphorylation, glycosylation, ubiquitination, SUMOylation, nitrosylation, methylation, acetylation, lipidation, and proteolysis, each contributing to the regulation of gene expression, immune responses, and pathogen-host interactions (Ramazi et al., 2020). In COVID-19 pathophysiology, phosphorylation, SUMOylation, and ubiquitination are particularly relevant due to their role in modulating STATs, IRFs, and NF-κB-mediated immune pathways.

Phosphorylation, one of the most prevalent PTMs, primarily occurs on serine, threonine, and tyrosine residues. This reversible modification is controlled by kinases, which transfer phosphate groups, and phosphatases, which remove them (Shi, 2009). By altering protein structure and charge, phosphorylation regulates enzymatic activity, molecular interactions, and intracellular trafficking, affecting immune signalling in response to viral infections. In COVID-19, phosphorylation plays a crucial role in activating STATs and IRFs, which are key regulators of interferon responses, while also modulating NF-κB activity, a central driver of inflammation.

### Phosphorylation

Phosphorylation, a reversible modification controlled by kinases and phosphatases, influences key immune regulators like STATs, IRFs, and NF-κB, which are essential in COVID-19 pathogenesis (**Figure 2**) (Glanz et al., 2021; Rincon-Arevalo et al., 2022; Farrokhi Yekta et al., 2024). By modifying serine, threonine, and tyrosine residues, phosphorylation activates signalling pathways that shape the immune response to SARS-CoV-2 (Shi, 2009).

In relation to COVID-19, phosphorylation enhances the activity of STATs, allowing their translocation to the nucleus and subsequent regulation of IFN signalling (**Table 1**). STAT1 and STAT2 phosphorylation facilitate the transcription of ISGs, which are crucial for antiviral defence (Znaidia et al., 2022). However, SARS-CoV-2 has evolved mechanisms to suppress STAT-mediated responses, reducing IFN production and enabling viral persistence (**Table 1**) (Cheng et al., 2023). Simultaneously, NF-κB activation, driven by phosphorylation, induces the expression of pro-inflammatory cytokines, including IL-6, TNF-α, and IFN-β, contributing to cytokine storms and tissue damage (Hariharan et al., 2021). Furthermore, IRF3 and IRF7 phosphorylation regulate IFN-α/β production, strengthening innate immunity, but the virus inhibits IRF activation, further dampening antiviral responses (Chen et al., 2013).

Variant-specific differences further refine this transcriptional landscape. The prototype strain robustly activates inflammatory TFs, including NF-κB, STAT1/3, and IRF3/7, resulting in enhanced cytokine output and severe immunopathology (Chen et al., 2013; Hariharan et al., 2021; Nishitsuji et al., 2022; Cheng et al., 2023). In contrast, Omicron variants exhibit reduced virulence but actively suppress STAT1 and IRF3 phosphorylation, dampening ISG transcription while sustaining NF-κB activity via ubiquitinated viral proteins such as NSP6 and ORF7a (Chen et al., 2013; Hariharan et al., 2021; Nishitsuji et al., 2022; Cheng et al., 2023). This preserved NF-κB signalling highlights a conserved pro-inflammatory strategy across strains.

In addition to canonical TFs, Omicron infection induces alternative regulatory pathways, notably Heat shock factor 1 (HSF1) activation. Phosphorylation of HSF1 at Ser326 promotes a stress-adaptive transcriptional response that may interact with NF-κB and STAT3 to modulate inflammation and cellular stress (Janus et al., 2022; Pauciullo et al., 2024). Furthermore, immune memory shaped by prototype exposure can be altered by Omicron reinfection, disrupting TF responsiveness and immune trajectory (Wei et al., 2025).

The long-term impact of TF dysregulation is evident in post-acute COVID-19 syndromes. Patients infected with the prototype strain are more susceptible to long COVID than those infected with Omicron, possibly due to sustained activation of pro-inflammatory TFs like NF-κB, STAT3, and IRF3/7, which contribute to chronic inflammation and tissue remodelling (Fernández-de-las-Peñas et al., 2022). One example involves COVID-19–associated kidney injury, where excessive STAT3 phosphorylation at Ser727, coupled with diminished Tyr705 activity, drives immune-mediated damage. NF-κB phosphorylation at Ser276 further amplifies inflammatory signalling, and STAT3-mediated ACE2 upregulation enhances viral entry in cytokine-enriched environments (Mokuda et al., 2020; Salem et al., 2022). Persistent activation of the IL-6/STAT3/ACE2 axis in pulmonary and synovial tissues has been linked to reinfection risk and fibrotic progression (Mokuda et al., 2020).

Therapeutically, modulating phosphorylation-driven TF activity holds promise. 6-O-angeloylplenolin (6-OAP) inhibits STAT3 phosphorylation, thereby reducing ACE2 expression and potentially limiting viral entry (**Table 2**) (Liang et al., 2022). Given the synergistic role of NF-κB and STAT3 in driving inflammation, dual-targeting strategies may offer more effective control of cytokine storms and restoration of immune balance (Fan et al., 2013). Additionally, enhancing IRF-mediated antiviral signalling could counteract SARS-CoV-2’s interferon suppression tactics.

**Table 2:** Therapeutic targets and agents modulating TFs in COVID-19.

Target TF/Pathway	Drug/Intervention	Mechanism
AHR	CH223191 and resveratrol	Reduction of immune suppression by limiting viral replication
NF-κB	Dexamethasone and curcumin	Pharmacological inhibitors that suppress NF-κB activation
JAK/STAT	Baricitinib and ruxolitinib	Suppress excessive inflammation and fibrosis.
HIF-1α	PX-478	Mitigation of hypoxia-induced immune dysregulation
NRF2	Not reported	Counteracts oxidative stress
PPARγ	Not reported	PPARγ trans-repression helps suppress NF-κB-driven inflammation and cytokine production
IRFs	Not reported	Enhance anti-viral responses and reinforce host defences

Overall, the intricate crosstalk among phosphorylation events and immune regulatory networks plays a decisive role in COVID-19 progression. Better understanding and targeted modulation of these pathways may improve antiviral efficacy, reduce immunopathology, and guide the development of tailored therapeutic interventions.

### SUMOylation and ubiquitination

SUMOylation and ubiquitination are fundamental post-translational modifications that dictate the activity of key transcription factors involved in immune regulation and oxidative stress responses during COVID-19. The interactions among PPARγ, NRF2, AHR, and NF-κB form a highly intricate regulatory network that governs inflammatory and anti-viral defences (**Figures 1**, **2**). SARS-CoV-2 strategically exploits these pathways to manipulate immune responses, enabling viral persistence while exacerbating pathological inflammation.

SUMOylation of PPARγ plays a critical role in suppressing excessive inflammatory signalling (**Table 1**). By recruiting co-repressors to NF-κB target gene promoters, SUMOylated PPARγ effectively limits the expression of pro-inflammatory cytokines, thereby contributing to immune homeostasis (Ghisletti et al., 2007). However, during severe COVID-19, PPARγ expression is significantly downregulated in lung monocyte-macrophages, leading to macrophage hyperactivation and an exacerbated cytokine response (Desterke et al., 2020). The overwhelming of SUMOylation mechanisms disrupts PPARγ’s inhibitory influence over NF-κB, causing unchecked inflammation and immune dysregulation.

NRF2, another pivotal transcription factor, undergoes continuous ubiquitin-mediated degradation by the KEAP1-CUL3 E3 ligase complex under normal physiological conditions. However, upon SARS-CoV-2 infection, oxidative stress inhibits KEAP1-dependent ubiquitination, allowing NRF2 to accumulate and activate cytoprotective pathways aimed at mitigating viral-induced oxidative damage (Kombe Kombe et al., 2024). While less characterized, SUMOylation is thought to fine-tune NRF2’s nuclear retention and coactivator interactions, further modulating the magnitude of the antioxidant response. The interplay between these modifications highlights potential therapeutic strategies aimed at enhancing NRF2’s activity to counteract oxidative lung injury associated with COVID-19.

AHR is tightly regulated by ubiquitination following ligand activation, ensuring precise control over gene expression (Farooqi et al., 2023). This transcription factor interacts dynamically with both NRF2 and NF-κB, exerting dual functions that either dampen inflammatory responses or enhance detoxifying gene expression depending on cellular conditions (**Figure 1**) (Wardyn et al., 2015). Although direct SUMOylation of AHR in the context of COVID-19 has not been conclusively demonstrated, studies on related nuclear receptors suggest that SUMO modification may contribute to its nuclear retention and transcriptional specificity (**Table 2**) (Xing et al., 2012). Such regulation has important implications for viral pathogenesis and immune resolution, particularly in the context of sustained inflammation triggered by SARS-CoV-2.

This transcriptional disruption extends beyond acute infection. In long COVID, sustained activation of key TFs such as STAT3 and NF-κB contributes to chronic inflammatory states. STAT3, frequently activated by IL-6 and associated with elevated ACE2 expression, may increase tissue vulnerability to reinfection and drive fibrotic remodelling (Mokuda et al., 2020). NF-κB remains persistently active in many long COVID profiles, maintaining low-grade inflammation and altering mitochondrial dynamics via MAVS SUMOylation (Li et al., 2021). At the same time, protective TFs such as NRF2 and PPARγ may be insufficiently activated, particularly when their PTM-mediated regulatory pathways are impaired. This may compromise antioxidant defences and the resolution of inflammation. AHR also participates in immune recalibration through ubiquitin-dependent regulation of detoxifying and inflammatory genes, though its long-term SUMOylation status in post-acute COVID-19 remains insufficiently explored.

Given the pivotal role of SUMOylation ubiquitination and epigenetic regulation in regulating these transcription factors, modulating these pathways presents promising therapeutic strategies to restore immune balance in COVID-19. Strategies aimed at enhancing NRF2 activity, reinstating PPARγ-mediated trans repression, or countering SARS-CoV-2-induced NF-κB hyperactivation hold significant potential for mitigating inflammation and tissue damage in infected individuals. By understanding these molecular interactions, novel therapeutic interventions can be developed to fine-tune immune responses and improve patient outcomes.

Additional epigenetic mechanisms, including DNA methylation, histone modifications and chromatin remodelling, also contribute to the regulation of AHR. These processes influence transcriptional accessibility and transcription factor binding affinity, shaping gene expression dynamics in response to infection and inflammation. Their roles in regulating AHR and related transcription factors during COVID-19 have been extensively reviewed by our group (Kgatle et al., 2021). As the current review focuses on transcription factor crosstalk and post-translational modifications in the context of COVID-19, a detailed discussion of these epigenetic pathways has been omitted to avoid redundancy.

## Therapeutic avenues

Future therapeutic strategies for COVID-19 must consider the complex interactions among described key TFs These regulators play essential roles in immune responses, cytokine signalling, oxidative stress defence, and metabolic adaptation. Their dysregulation contributes to disease severity, making them prime targets for intervention. By modulating TF activity, researchers aim to restore immune balance, mitigate hyper-inflammation, and enhance antiviral defences.

AHR, with its dual role in immunosuppression and inflammatory cytokine production, remains a promising therapeutic target. AHR antagonists like CH223191 and resveratrol may help reduce immune suppression while limiting viral replication (**Table 2**). Additionally, the interplay between AHR and NRF2 presents an opportunity to counteract oxidative stress. NRF2 activation, disrupted in severe COVID-19 cases, has been proposed as a strategy to enhance antioxidant defences and reduce tissue damage. Targeting these pathways could improve immune resilience and overall patient outcomes.

NF-κB is a major driver of cytokine storm syndrome, contributing to excessive inflammation and disease progression. Pharmacological inhibitors such as dexamethasone and curcumin have been shown to suppress NF-κB activation, reducing uncontrolled immune responses (**Table 2**). Similarly, interventions targeting the JAK/STAT pathway, including baricitinib and ruxolitinib, may suppress excessive inflammation and fibrosis (**Table 2**). Modulating these pathways holds potential for improving recovery in patients with severe COVID-19.

Hypoxia-driven inflammation, largely mediated by HIF-1α, represents another therapeutic avenue. PX-478, a known HIF-1α inhibitor, could help mitigate hypoxia-induced immune dysregulation, thereby reducing lung damage and systemic complications (**Table 2**). Additionally, IRF3/IRF7 activation via IFN-β therapy may enhance anti-viral responses, reinforcing host defences against SARS-CoV-2 replication.

The metabolic regulators NRF2 and PPARγ also provide therapeutic opportunities. NRF2 counteracts oxidative stress, while PPARγ trans repression helps suppress NF-κB-driven inflammation and cytokine production. Pharmacological interventions designed to enhance NRF2 activity or activate PPARγ may restore metabolic equilibrium and immune control as observed in COVID-19. Given the observed disruption of PPARγ function in severe COVID-19 cases, strategies aimed at reinstating its role in immune modulation could significantly impact disease management.

Overall, advancing targeted TF modulation presents a promising approach to mitigating severe COVID-19 symptoms and long-term complications. By refining therapeutic interventions based on TF crosstalk, researchers may develop more effective treatments for controlling immune dysregulation, preventing tissue damage, and improving patient recovery. Continued research into TF regulatory mechanisms will be essential in shaping future antiviral and immunomodulatory strategies.

## Conclusion and future perspectives

The classification and regulatory interactions of key TFs in COVID-19 reveal the intricate molecular networks that govern immune regulation, inflammation, and metabolic adaptation. The disruption of these key TFsby SARS-CoV-2 severely alters immune regulation, extends viral persistence, and accelerates disease progression.

These transcription factors fulfil distinct roles in disease modulation. Pro-inflammatory regulators such as NF-κB, STAT3, and AHR drive cytokine storms and immune hyperactivation, intensifying clinical severity. In contrast, immune-modulatory TFs, including NRF2, PPARγ, and ATF3, help suppress excessive inflammation, though SARS-CoV-2 weakens their protective function. Additionally, interferon-related TFs (IRFs and STATs) are essential for anti-viral defence, yet the virus actively disrupts their signalling pathways, impairing immune responses. Stress-adaptive regulators, such as HIF-1α and NRF2, further influence hypoxia response and metabolic reprogramming, which contribute to disease progression.

The interconnections among TFs, shaped by shared signalling cascades, metabolic interactions, and post-translational modifications (SUMOylation, ubiquitination, phosphorylation), illustrate the complexity of transcriptional regulation in COVID-19. SARS-CoV-2 manipulates these molecular interactions to sustain inflammation while suppressing antiviral defences, reinforcing immune imbalance.

Targeted therapeutic strategies leveraging TF modulation present a promising direction for intervention. Suppressing NF-κB activation through dexamethasone, optimizing JAK-STAT signalling with baricitinib, enhancing NRF2 activity to counteract oxidative stress, and inhibiting AHR-mediated immune suppression could collectively improve patient outcomes. Additionally, mitigating hypoxia-induced inflammation via HIF-1α inhibitors and reinforcing interferon pathways through IFN-β therapy may help restore immune balance and improve recovery.

This review provides a comprehensive foundation for understanding transcription factor classification and regulatory interactions, offering insights into host-pathogen dynamics, immune adaptation, and therapeutic interventions. Advancing research into TF-driven transcriptional regulation and PTM-mediated interactions will be crucial for refining precision-targeted therapies that effectively address immune dysregulation, inflammation, and metabolic disruptions caused by SARS-CoV-2. By strategically modulating these pathways, future treatments may successfully restore immune resilience, minimize inflammatory damage, and enhance long-term recovery outcomes. Understanding the molecular mechanisms underlying transcriptional responses in COVID-19 will pave the way for next-generation antiviral strategies, shaping the future of personalized immunotherapy and precision medicine.
